# A Case of Gastrointestinal Krukenberg Tumor Initially Suspected as Primary Ovarian Cancer and the Implications for Management

**DOI:** 10.7759/cureus.112822

**Published:** 2026-07-16

**Authors:** Nabarun Sidhu, Brandon M Le, Ethan L Doan, Kevin T Dao, Wilbur Montana

**Affiliations:** 1 Internal Medicine, University of California, Los Angeles Kern Medical, Bakersfield, USA; 2 Hematology and Medical Oncology, University of California, Los Angeles Kern Medical, Bakersfield, USA

**Keywords:** gastric adenocarcinoma, gastrointestinal primary, krukenberg tumor, ovarian metastatic cancer, primary ovarian cancer

## Abstract

Krukenberg tumors are an uncommon metastatic malignancy most commonly originating primarily from the gastrointestinal tract, particularly the stomach, colon, and/or rectum. Due to the rarity of these malignancies, most patients are believed to have primary metastatic ovarian malignancy instead. Here, we present the case of a patient who was presumed to have ovarian metastatic cancer; however, pathology results showed that the malignancy was gastrointestinal primary in nature. In this instance, intraoperative findings and immunohistochemical profiling played a crucial role in differentiating a Krukenberg tumor with a gastrointestinal primary versus ovarian metastatic cancer. In this case, proper diagnosis resulted in a change of management, highlighting that even the most unlikely differentials should still be considered.

## Introduction

Krukenberg tumor is a metastatic ovarian cancer that generally originates in the gastrointestinal tract. This form of cancer consists of mucin-secreting signet-ring cells with an incidence of 1% to 21% that can spread from various locations, such as the colon, pancreas, breast, bowel, appendix, and genitourinary system (urinary and reproductive systems) [1]. These tumors usually account for a small proportion of all ovarian malignancies and generally are bilateral in nature [1,2]. Radiologically, these malignancies can present as cystic, solid, or a combination of both [1]. Clinically, patients often present with non-specific symptoms such as pelvic pain and abdominal distension [1]. Hence, certain gastrointestinal tumor markers, such as CK20 and CDX2 positivity with CK7 negativity, can play a crucial role in confirming the diagnosis [2]. As a result, a definitive diagnosis requires immunohistochemical studies along with histopathological examination.

On the other hand, primary ovarian malignancies can arise from sex cord-stromal tissue, ovarian surface epithelium, or germ cells. These cancers are the leading cause of death from a gynecological malignancy as well as the second most common pelvic malignancy [3]. Epithelial tumors, particularly serous and mucinous carcinomas, are the most common [1,4]. Unfortunately, epithelial tumors are frequently diagnosed at an advanced stage due to non-specific symptoms such as abdominal distension, pelvic pain, or gastrointestinal complaints [1,4]. Thus, imaging modalities such as ultrasound and computed tomography (CT), along with tumor markers, particularly cancer antigen-125, are instrumental in the initial diagnosis. However, as with most malignancies, diagnosis still relies on histopathology.

Despite having similar clinical presentations, the differentiation between primary ovarian carcinoma and metastatic gastrointestinal cancer to the ovaries is central to guiding management as well as estimating the prognosis. Therefore, we present the case of a patient who was believed to have a primary ovarian cancer. However, after surgical interventions, immunohistochemical studies, and histopathological examination, it was determined that the patient had metastatic gastrointestinal cancer.

## Case presentation

A 58-year-old female was transferred from another facility for a higher level of care due to a suspected ovarian malignancy that was seen via ultrasound at the other facility. On arrival, the patient stated that she initially presented to another facility due to getting up too fast and falling, but denied any loss of consciousness. The patient stated that her primary concern was her abdomen slightly increasing in size. However, she noted that she was not sure when her symptoms first began. She also reported some weight loss, loss of appetite, bloating, and constipation, but denied any other symptoms at this time. Furthermore, she denied any family history of cancer.

**Figure 1:**
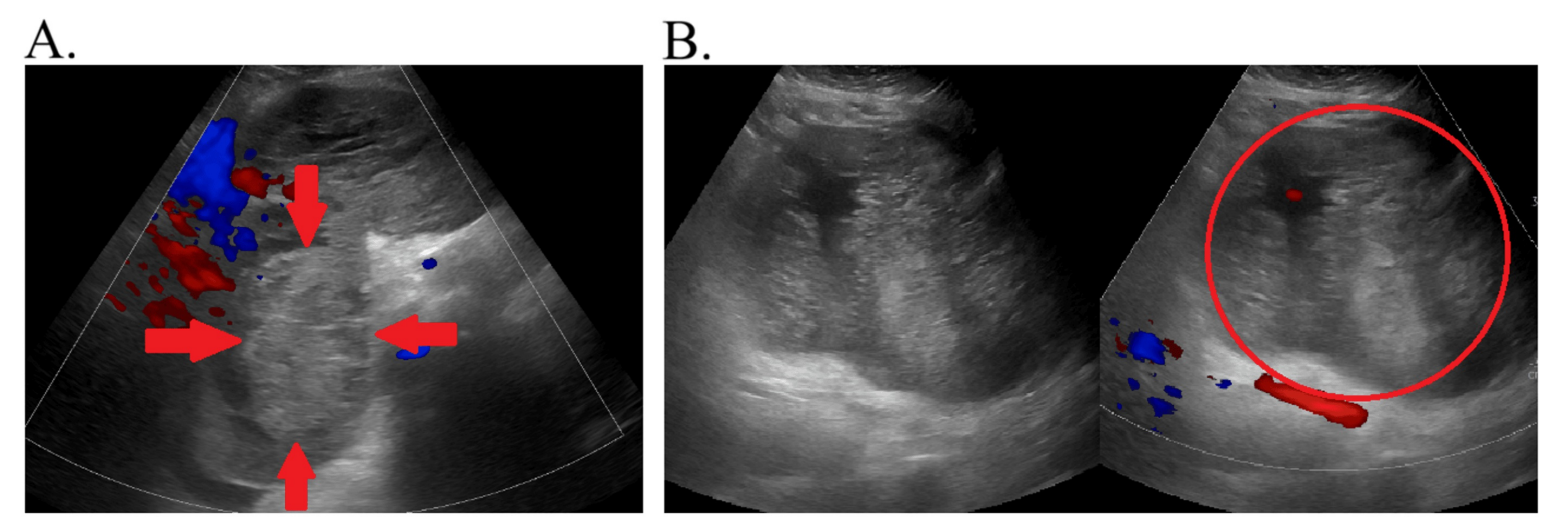
Ultrasound of the abdomen. (a) Ultrasound imaging performed at the prior facility showing an abdominal mass indicated by the arrows. (b) Ultrasound imaging performed at our facility showing an abdominal mass indicated by the circle.

**Figure 2:**
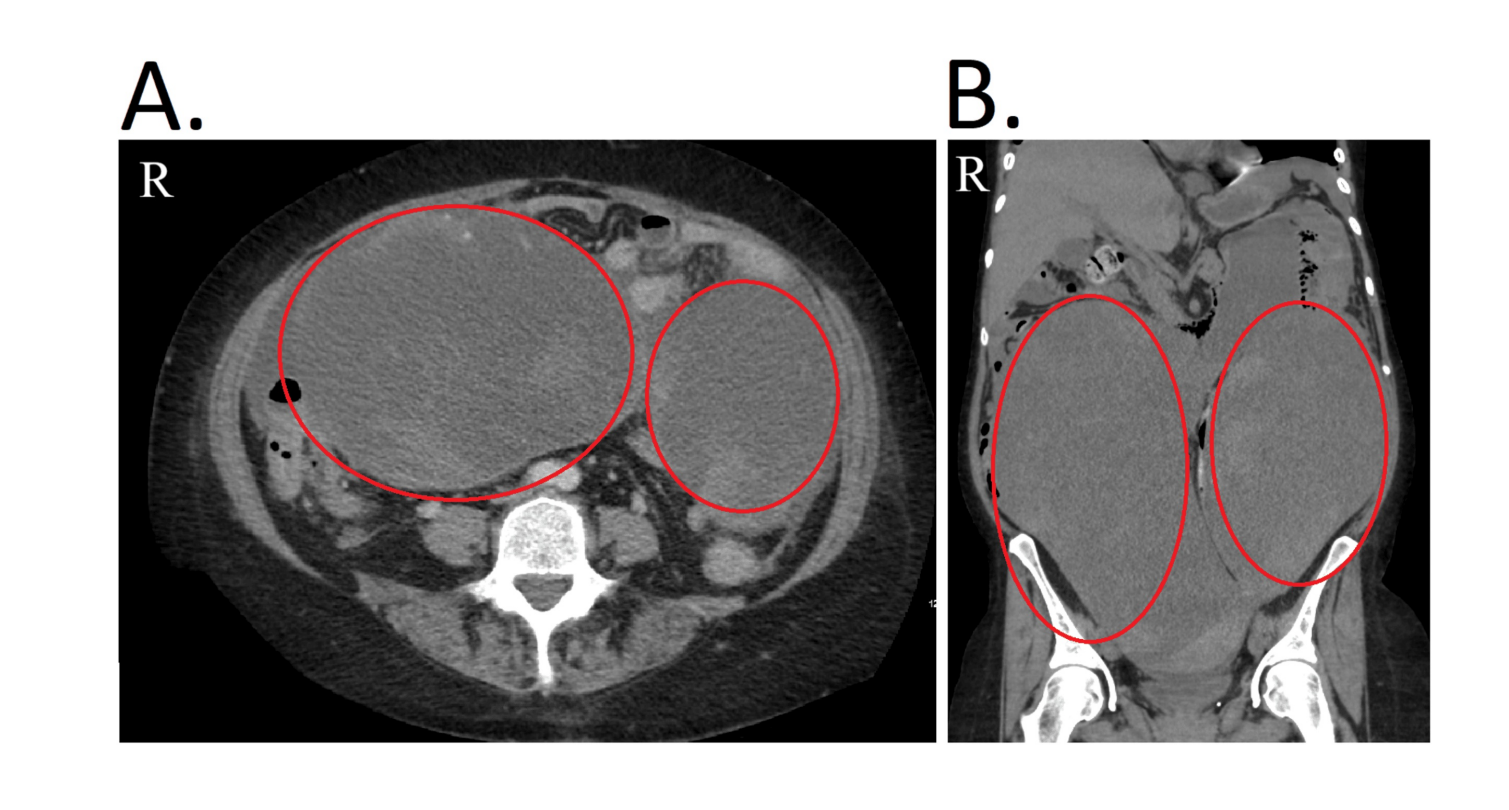
Computed tomography (CT) of the abdomen and pelvis without contrast. (a) CT transverse view shows approximately right lower abdominal well-defined heterogeneous enhancing soft-tissue mass measuring 12.7 × 16.5 × 20.9 cm and left lower abdominal well-defined heterogeneous enhancing soft tissue mass measuring 13.3 × 8.6 × 13.2 cm noted by the red circles. (b) CT coronal view of Figure 2a.

On physical examination, her vitals were noted to be unremarkable; however, the patient’s abdomen was distended and tense with no tenderness on palpation, and bowel sounds were difficult to appreciate. The patient had a small laceration on her forehead due to the fall and +1 pitting edema at her feet; however, the rest of the physical examination was unremarkable. The patient declined a pelvic examination. A complete blood count and basic metabolic panel were performed (Table 1). Ultrasound imaging, which was repeated at our facility, depicted a heterogeneous mass measuring approximately 12.7 × 16.5 × 20.9 cm (Figures 1a, 1b). A CT scan without contrast was performed owing to concerns about acute kidney injury (Figures 2a, 2b).

**Table 1:** Blood count and basic metabolic panel with tumor markers.

Parameter	Value	Reference range
Sodium	124 mmol/L	136–145 mmol/L
Potassium	4.5 mmol/L	3.5–5.1 mmol/L
Chloride	91 mmol/L	98–107 mmol/L
Calcium (corrected)	10.2 mg/dL	8.5–10.1 mg/dL
Magnesium	2.6 mg/dL	1.8–2.4 mg/dL
Phosphorus	7.2 mg/dL	2.5–4.9 mg/dL
Creatinine	3.52 mg/dL	0.51–0.95 mg/dL
Glomerular filtration rate	13 mL/minute/1.73 m²	>60 mL/minute/1.73 m²
Albumin	1.5 g/dL	3.4–5 g/dL
Alanine transaminase	23 U/L	13–61 U/L
Aspartate transaminase	57 U/L	15–37 U/L
Direct bilirubin	0.3 mg/dL	0–0.2 mg/dL
Total bilirubin	0.7 mg/dL	0–1 mg/dL
White blood count	26.5 × 10³/µL	4.5–11 × 10³/µL
Hemoglobin	8.1 g/dL	13.2–17.4 g/dL
Mean corpuscular volume	82.1 fL	80–98 fL
Platelet	597 × 10³/µL	150–450 × 10³/µL
Cancer antigen-125	313 U/mL	<35 U/mL
Cancer antigen-19-9	191 U/mL	<34 U/mL
Human epididymis protein 4 ovarian cancer antigen	9,697 pmol/L	<140 pmol/L
Inhibin A	7 pg/mL	<2.1 pg/mL
Inhibin b	<10 pg/mL	<10 pg/mL

Nephrology was consulted, and it was believed that the patient’s underlying malignancy could contribute to her declining function, and management was recommended accordingly. Gynecology and Oncology were consulted, and the patient underwent an exploratory laparotomy, bilateral salpingo-oophorectomy with excision of bilateral adnexal masses, appendectomy, omentectomy, small bowel resection, and diverting end ileostomy. Pathology was sent; however, the patient became hypotensive, most likely due to hypovolemia, with difficulty being extubated. Thus, she was admitted to the intensive care unit (ICU).

**Figure 3:**
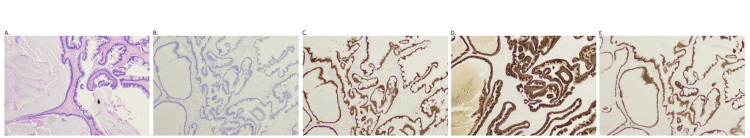
Pathology reports. (a) Hematoxylin and eosin stain. (b) CK 7 is negative based on the lack of brown staining. (c) CDX2 is positive based on brown staining which is a common gastrointestinal marker but can be an ovarian marker but is not specific. (d) CK20 is positive based on the brown staining. (e) SATB2 is positive based on brown staining. Immunohistochemical staining was done and tumor cells were noted to be CK7-, CK20+, PAX-, ER-, PR-, and SATB2+. The positivity for SATB2, CDX2, and CK20 suggests metastatic mucinous adenocarcinoma from gastrointestinal primary, particularly lower gastrointestinal tract.

Pathology then confirmed mucinous metastatic carcinoma from a lower gastrointestinal primary tumor from all resected specimens (Figures 3a-3e). A repeat CT of the abdomen and pelvis was performed with contrast after the patient’s kidney function had improved, which showed multiple interval postsurgical changes but was otherwise unremarkable. The patient was eventually extubated with a port-a-cath placed.

Medical oncology devised a plan to start chemotherapy four weeks after the surgery due to concerns of wound healing in the setting of extensive surgery. She was discharged, and a follow-up magnetic resonance imaging of the abdomen and pelvis was pending, which would help locate any residual tumors and help with staging.

However, one week after the patient was readmitted due to concerns of postoperative wound infection, she was started on intravenous (IV) antibiotics and readmitted. She was noted to have extensive necrotic wounds of the bilateral buttocks and thighs. General surgery was consulted with plans to take the patient to the operating room with general surgery for sharp excisional debridement of the bilateral thighs and wound vacuum placement on the left hip. However, the patient continued to decompensate and was noted to have a postprocedural intra-abdominal abscess. A drain was placed, and the patient continued on IV piperacillin-tazobactam 4.5 g every eight hours with IV vancomycin, with cultures pending. However, the patient had a long discussion with the primary care team and decided that she would like the drain removed and did not want to undergo any further management, such as chemotherapy, and would prefer palliative/hospice care. Palliative care was consulted, and the patient was shortly discharged home with hospice care.

## Discussion

Ensuring proper differentiation of primary ovarian malignancies from gastrointestinal metastatic malignancy to the ovaries is critical, as the management and prognosis differ considerably. In primary ovarian malignancies with metastasis, the standard of care generally includes cytoreductive surgery aiming for optimal debulking, followed by platinum-based chemotherapy [3,5]. In some instances, hyperthermic intraperitoneal chemotherapy can also be considered during episodes of cytoreductive surgery after neoadjuvant chemotherapy [5,6].

On the other hand, treatment for Krukenberg tumors as a gastrointestinal primary varies depending on whether they are gastric or colorectal. In the majority of cases, gastric cancers account for approximately 70% of cases, whereas colorectal cases account for approximately 15-20% of gastrointestinal Krukenberg cancers [2,4,6]. Krukenberg tumors with gastric adenocarcinoma primary generally depend on certain biomarkers (HER-2 and CLDN18.2) and whether the malignancy can be resected [7]. Some studies have shown that oophorectomy plus chemotherapy leads to improved survival in conjunction with chemotherapy alone [6,8]. The same studies have reported an approximate median overall survival of 19-24.6 months with surgical intervention along with chemotherapy in comparison to 11.8-14.3 months with only chemotherapy [6,8].

In Krukenberg tumors with colorectal cancer as the primary, the guidelines follow the colorectal cancer guidelines, with various genetic factors coming into play, such as *KRAS*/*NRAS*/*BRAF* mutations [9,10]. Similar to primary ovarian malignancies, patients with Krukenberg tumors from colorectal cancer have been shown to have an improved overall survival when undergoing cytoreductive surgery with oophorectomy. A meta-analysis, which included 12 cohort studies and 1,031 patients, noted an improved overall survival with R0 resection and metachronous metastasis associated with better outcomes [11]. However, it should be noted that patients with ascites and peritoneal carcinomatosis had worse survival [11,12]. Overall, complete cytoreduction along with systemic chemotherapy generally depicts a median survival of approximately 35 months [12].

Determining whether the patient has primary ovarian cancer or a Krukenberg tumor with gastrointestinal origin is key, as such a distinction helps guide management and prognosis. As such, there are some methods to help distinguish primary ovarian cancer and Krukenberg with gastrointestinal origin. Regarding radiological findings, gastrointestinal metastatic Krukenberg tumors tend to be bilateral, whereas primary ovarian cancers can be either bilateral or unilateral, depending on whether they are high-grade serous ovarian carcinomas or primary mucinous ovarian carcinomas [13-15]. Tumor size can also help determine if the patient has primary mucinous ovarian carcinomas, which are usually greater than 10 cm and are on average 16.4 cm, while metastases tend to be smaller, which makes the diagnosis of primary mucinous ovarian carcinomas more likely. Concerning immunohistochemistry, ovarian cancers tend to depict CK7+/SATB2-, whereas colorectal cancers are CK7-/SATB2+ [5,13,16,17]. These details were one of the primary reasons why our patient was suspected of having a Krukenberg tumor with a lower gastrointestinal primary (Figures 3a-3e).

Our patient was also noted to have some complications that resulted in her being transferred to the ICU; however, after she had stabilized, the patient was able to be discharged with follow-up and pending chemotherapy treatment. Unfortunately, she had developed infection-related complications. This also raises a key point regarding prognosis. Many studies have shown that the prognosis of patients differs greatly depending on the underlying primary malignancy. Despite having similar clinical presentations, the median survival for metastatic ovarian cancer is approximately 36-57 months with currently available treatment, whereas Krukenberg tumors from gastric cancer have a median survival of 13-24.6 months, and colorectal Krukenberg tumors that are surgical candidates have an approximate median survival of 35 months [5,8,12].

## Conclusions

In patients with suspected primary ovarian malignancies, histopathological studies as well as immunohistochemical examinations are crucial in determining management. Within the limitations of this case presentation, the possibility of an uncommon malignancy should be noted. This is particularly true as rare malignancies can have very similar presentations to more common cancers. Despite ovarian malignancy being the initial diagnosis in our patient, based on likelihood and presentation, the chance of developing a rare Krukenberg tumor is still possible, as in this case. Therefore, early recognition is essential for accurate diagnosis and appropriate oncologic management.
